# Influence of Overweight and Obesity on Bone Remodeling during Pregnancy

**DOI:** 10.21203/rs.3.rs-6606324/v1

**Published:** 2025-06-02

**Authors:** Ana Carolina Ariza, Eduardo Ortiz-Panozo, Héctor Lamadrid-Figueroa, Ángel Santiago, Juan Tamayo-Orozco, Mauricio Hernández-Ávila, Howard Hu, Adrienne S. Ettinger, Martha M. Téllez-Rojo, Karen E. Peterson, Deborah J Watkins, Marcela Tamayo-Ortiz

**Affiliations:** National Institute of Public Health; National Institute of Public Health; National Institute of Public Health; National Institute of Public Health; Accessalud; Mexican Social Security Institute; University of Southern California; Rutgers University; National Institute of Public Health; University of Michigan School of Public Health; University of Michigan School of Public Health; Columbia University

**Keywords:** body mass index, obesity, bone remodeling, pregnancy

## Abstract

**Background:**

The prevalence of maternal overweight and obesity during pregnancy continues to increase. Less is known on how this can influence bone health. The aim of this study was to evaluate the association between body mass index (BMI) and bone remodeling (BR) during pregnancy.

**Material and Methods:**

We evaluated 579 pregnant women participants of the ELEMENT cohort in Mexico City. We used mixed effects models to analyze the association between baseline BMI (normal, overweight, obesity) and trimester-specific measurements of urinary N-telopeptides (NTx, nM BCE/mM creatinine), radius axial quantitative bone ultrasonography speed-of-sound z-score (Z-SOS) in a subsample of 429 women and bone specific alkaline phosphatase in plasma in a subsample of 143 women. Models were adjusted for age, number of pregnancies, pregnancy weight gain, breastfeeding history, calcium, phosphorus, magnesium, and vitamin D intakes.

**Results:**

39% of women were overweight and 15% were obese in the first trimester of pregnancy. Compared to normal weight women, overweight and obese women had lower Z-SOS (b=−0.28, 95%CI: −0.49, −0.08 and b=−0.57, 95%CI: −0.87, −0.27, respectively), this difference seemed to be greater between 25 and 35 weeks of gestation.

**Conclusions:**

Our study found an attenuated bone speed of sound among overweight and obese pregnant women relative to their normal weight counterparts indicating more active bone remodeling. We provide evidence of women’s skeletal health in a gestational-obesogenic setting, follow-up of these women will provide insight to the long-term relation between pregnancy and midlife bone health.

## Background

During pregnancy, several metabolic adaptations take place in order to support fetal bone growth and calcification.^1^ Bone is made of both organic (e.g., collagen) and inorganic (e.g., mineral) components.^2^ Bone remodeling (BR) refers to a continuous process of replacement of mature bone clusters through resorption coupled with protein matrix synthesis and a subsequent mineralization process. A BR unit is composed of osteoclasts and osteoblasts-lineage cells that are strongly influenced by hormones present in pregnancy.^3^ Therefore, maternal BR is highly active during gestation, and due to an increase in maternal bone resorption, particularly in the third trimester.^4^

Worldwide, overweight and obesity prevalence in women of reproductive age and during pregnancy has increased over the previous decades, with women starting their pregnancy with excess weight.^5-8^ A recent systematic review and meta-analysis confirmed this increasing trend, estimating the current global prevalence of maternal obesity at 20.9% (95% CI 18.6 to 23.1%), with projections to reach 23.3% (95% CI 20.3 to 26.2%) by 2030. However, this prevalence varies across regions, being higher in North America and Australia/Oceania, and lower in Asia. Authors also report that 17% of pregnancies globally and 25% in North America are complicated by obesity.^9^ Obesity is characterized by a subclinical and chronic inflammatory state that affects several physiological processes including bone metabolism.^10^ Inflammation may influence BR through the synthesis of adipokines such as IL-6 and TNF-α, which regulate osteoclast activation. This inflammatory state has effects on calcium and phosphorus plasma levels by altering the regulation of parathyroid hormone (PTH) and levels of 25-hydroxyvitamin D (the inactive form of Vitamin D). PTH stimulates the release of calcium in an indirect process through osteoclasts which ultimately leads to bone resorption,^11^ while the active form of vitamin D (1,25-hydroxyvitamin D) enhances osteoclastogenesis.^12^ Each stage of osteoclastic activation is followed by a coupling of bone formation processes, such as mineralization of the same quantity of organic matrix destroyed, hence the term remodeling.^11^

Some clinical and epidemiological studies in adults with obesity have suggested an alteration in the regulation of BR resulting in an increase in bone mineral density with a decrease in turnover, although the mechanism is still unknown.^13-16^ However, chronic inflammation from overweight and obesity could contribute to decreased BR in pregnancy.^17,18^ Studies on BR during pregnancy are limited, so there is little information regarding the effect of excess pregnancy weight on BR and bone turnover biomarkers. Furthermore, few studies have repeated measures during pregnancy of biomarkers of BR and bone density. This study aimed to evaluate the association between excess weight and BR including each pregnancy trimester. We hypothesized that women with obesity would have lower BR and bone turnover measurements during pregnancy compared to women with normal or overweight BMI.

## Methods

### Participants and study design

This study included women from the Early Life Exposures in Mexico to Environmental Toxicants (ELEMENT) cohort.^19^ Pregnant women attending prenatal care clinics that belong to the Mexican Institute of Social Security were recruited between 2001 and 2005. Women were originally participating in a randomized trial that evaluated the effect of 1,200 mg calcium carbonate daily supplementation on lead mobilization from bone during pregnancy, the trial has been described in detail and its results published elsewhere previously.^20,21^ Diagnosis of mineral metabolism conditions, preeclampsia, gestational diabetes or a high-risk pregnancy, renal or cardiac diseases, infections, personal or family history of kidney stones, steroid treatment or the intention not-to-breastfeed were considered as exclusion factors.

Eligibility criteria included less than 14 weeks pregnancy and the intention to maintain residence in Mexico City for at least 5 years. Of the 670 women who agreed to participate, 579 women had at least one measurement of creatinine-adjusted urinary concentrations of N-Telopeptides of type I collagen (NTx) during pregnancy, while a subsample of 429 women had at least one measurement of appendicular, axial bone ultrasonography (speed of sound, SOS) at the non-dominant distal radius, and finally a subsample of 143 women had at least one measurement of bone-specific alkaline phosphatase in plasma. Women with missing information on covariates were excluded from the final models, with analytical samples therefore of 579, 414 and 143 for NTX, BAP and SOS models, respectively.

All the procedures in this study were conducted according to the guidelines of the Declaration of Helsinki. The protocol was approved by the Research and Ethics Committees of the National Institute of Public Health in Mexico, the Mexican Social Security Institute, the Brigham & Women's Hospital and the Harvard School of Public Health. All participants signed an informed consent at enrollment.

### Body Mass Index

Trained and standardized personnel obtained weight and height measurements once in every pregnancy trimester study visit by using conventional and internationally accepted protocols.^22,23^ Body mass index (BMI; weight in kg divided by squared height in m) was categorized as normal weight (BMI<25 kg/m^2^), overweight (BMI between 25 and 29.9 kg/m^2^) and obesity (BMI ≥30 kg/m^2^) according to the WHO guidelines.^24^ We used the first trimester BMI to define weight group category. Pregnancy weight gain was calculated as the difference in weight between each of the study visits, concurrent with the bone measurements.

### Bone Ultrasound

Attenuation of speed of sound was measured at the distal radius by quantitative ultrasound (Sunlight Omnisense 8000P, BeamMed Ltd, Tel Aviv, Israel). This anatomical region was chosen because it allows repeated measurements through the longitudinal axis of the bone. This equipment has 1 transducer, with one transmitter and one receiver, which produce acoustic waves at an average frequency of 1.25 MHz. To calculate the speed of sound (SOS), the propagation time between transmitter and receiver was measured following the manufacturer’s protocol. This measurement was repeated at each of the pregnancy trimester study visits, allowing us to follow a complete process of BR (from start to end of osteoid production) which is completed in approximately 130 days. A total of 974 measurements were obtained for women across study visits (women with 1 measurement n=98, 2 measurements n= 117, 3 measurements n=214). Importantly, the bone ultrasound measures the attenuation in meters/second of SOS in the axial axis in a hemicylinder of the appendicular bone. Smaller measurements indicate more presence of collagen and non-mineralized tissue, hence, increased bone remodeling.^25^ Distal radius SOS Z-scores (Z-SOS) for BR were obtained automatically using manufacturer reference values. Z-score relates the SOS value to an age-matched database of the same sex and ethnic origin as the patient. The Z-score value is the number of standard deviations by which the current patient's SOS value exceeds or falls below the mean for the age- and gender-matched group.^26^

### Urinary Crosslinked N-telopeptides of Type I Collagen (NTx) and Bone-specific alkaline phosphatase (BAP)

Urinary crosslinked N-telopeptide (NTx) is a type I collagen breakdown product produced by osteoclastic digestion of bone collagen that serves as a biological marker of bone resorption, with higher values indicating higher BR.^27-29^ NTx was determined in 1,641 urine samples from 579 women following the method as previously described.^21^ Briefly, urine samples were analyzed with a commercial competitive-inhibition enzyme-linked immunosorbent assay (ELISA) (Osteomark; Ostex International; Seattle, WA). NTx concentrations were expressed as nanomoles of bone collagen equivalents normalized to creatinine (nmol BCE/mmol creatinine). The intra- and inter-assay coefficients of variation were below 10%. Bone-specific alkaline phosphatase (BAP) was measured in plasma stored at −70°C from a subset of participants (N = 143) using the Ostase^®^ BAP immunoenzymetric assay (Immunodiagnostic Systems Inc., Fountain Hills, AZ). BAP levels reflect the metabolic status of osteoblasts and, thus, serve as an indicator of bone formation.^30,31^

### Covariates

Age at recruitment, years of education, age at first pregnancy, number of pregnancies and total months of breastfeeding (i.e., including previous pregnancies or none for primigravidae women) were obtained from baseline questionnaire.

Daily intakes of calcium, phosphorus and magnesium were assessed at each visit using a semi-quantitative food frequency questionnaire designed to estimate the usual dietary intake during the previous month. The questionnaire was validated for women residing in Mexico City^32^ and included specific questions for pregnancy, such as any additional use of dietary supplements. For women assigned to the 1,200 mg calcium carbonate (480 mg of elemental calcium) supplement arm of the clinical trial, we added the estimated actual consumption of calcium based on compliance with the assigned treatment to daily dietary intake. Compliance for this trial was high and has been previously reported.^20^

### Statistical Analysis

Means, standard deviations and percentages were calculated for each category of pregnancy BMI. Differences across BMI categories were tested by Kruskal-Wallis tests. Mixed effects linear models were used to evaluate the associations between pregnancy BMI categories and repeated measures of Z-SOS, urinary NTx and blood BSAP. Log-transformed values were used for NTX and BAP due to the skewed distribution of these biomarkers. Models were adjusted for age at recruitment (years), education (years), age at first pregnancy (years), number of previous pregnancies, total breastfeeding (months), pregnancy weight gain between each study visit (kg), total intake of calcium (mg) (including supplementation), phosphorus (mg), magnesium (mg) and vitamin D (ng), and a 4^th^ degree polynomial of gestational age in weeks (selected by model’s best fit). For primigravidae women, total breastfeeding was defined as zero months and age at first pregnancy as age at their last menstrual period. Pregnancy weight gain (kg) was defined as the difference of weight between each study visit and baseline values.

To explore whether BR marker trajectories differed by pregnancy BMI categories, interaction terms between BMI categories and the fourth-degree polynomial of gestational age (weeks) at each study visit (1^st^ trimester visits ranged 6-18, 2^nd^ trimester 19-28, and 3^rd^ trimester more than 28 weeks) were included in mixed effects models. All statistical analyses were performed using Stata for Windows, version 18.0 (StataCorp LP, College Station, Texas).

## Results

Of the 579 women evaluated in this study, 39% and 15% met the criteria for being overweight and being obese at the first trimester visit, respectively. The mean (SD) age was 26.4 (SD=5.5) years. Across BMI categories, age, number of pregnancies, and weight gain were significantly different (Table 1). Women who were overweight or obese were older than women with normal weight (p<0.001). Pregnancy weight gain was lower for women who were overweight or obese, respectively (p<0.001). Moreover, the number of previous pregnancies was higher for women with overweight or obesity (p<0.008).

In mixed models, we observed that compared to normal weight women, women who were overweight or obese had a mean Z-SOS score 0.28 standard deviations lower (95%CI: −0.49, −0.08) and 0.57 lower (95%CI: −0.87, −0.27), respectively. No significant association with NTX or BAP values was found, although in both cases coefficients tended to be negative relative to those with normal weight, especially in the obese group (Table 2).

**Figure 1:**
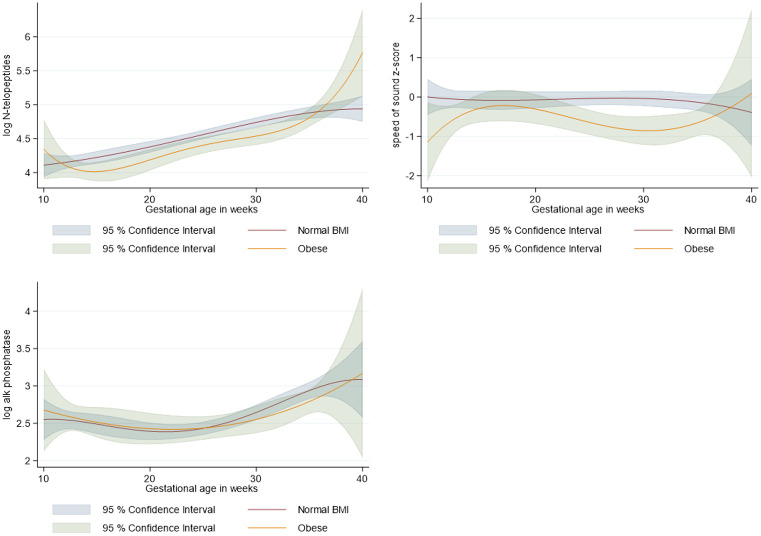
Fitted values and 95% confidence Intervals of bone turnover indicators during pregnancy by BMI categories at first trimester visit. A. Logarithm of Urinary N-telopeptides of type I collagen (nM BCE/mM creatinine); women=579, observations=1629. B. Radius speed-of-sound Z-scores; women=414, observations=922. C. Logarithm of plasma Bone-Specific Alkaline Phosphatase; women=143, observations=354). Adjusted for age at recruitment, education, age at first pregnancy, number of pregnancies, total breastfeeding, pregnancy weight gain at each trimester visit, total calcium intake, dietary intake of phosphorus, magnesium, and vitamin D.

Figure 1 shows the estimated trajectories of BR indicators throughout pregnancy by 1^st^ trimester BMI categories. In women who started pregnancy with a normal weight, a slight increase in Z-SOS scores from first to second trimester was observed, with a shift that showed a slight decrease in Z-SOS at the end of pregnancy. In contrast, women that started their pregnancy obese had lower Z-SOS scores in comparison with women with normal weight, especially between gestational age 25 to 35 weeks (Figure 1B). NTx increased in all BMI categories from first to third trimester, while BAP decreased slightly up to week 25 and increased back afterwards. NTx concentrations tended to be lower in the obese group in comparison with women with normal weight, although at no point confidence bands ceased to overlap, similar to what was observed in the BAP models (Figures 1A and 1C).

## Discussion

In this study with repeated concurrent measurements of radius Z-SOS scores, urinary NTx and plasma BAP across pregnancy, we observed that women with excess weight showed a different pattern of bone remodelling in contrast to women with normal weight. This has important implications due to the increasing number of women worldwide starting their pregnancy with overweight or obesity. In our study, more than half of participating women were overweight or obese in the first trimester of pregnancy which is consistent with national and international trends.^5-7,33^

Our findings reflect the expected higher demand of mineral and increased bone remodelling as pregnancy advances;^34,35^ lower SOS measures reflect increased bone matrix activity and a higher collagen content. As compared to normal-weight women, women with excess weight had lower Z-SOS scores at first trimester, which decreased further in second trimester, and this pattern was maintained until close to the end of pregnancy. This decreasing pattern was even more pronounced among obese women. Interestingly, relative to normal weight women, NTx values tended to be lower in overweight and obese women. This can be reflecting the BMD and obesity paradigm. Several research groups have studied bone remodelling or bone mineral density (BMD) associated with obesity. Evidence suggests that, in adolescents or adults with obesity, cortical bones show more stiffness, while changes in BMD and urinary biomarkers suggest an increased mineralization.^8,13,14,36^ This increase could be compensatory due to mechanical overload but few studies have evaluated bone remodelling and obesity during pregnancy.

Two studies of BMD measured with DXA scans early in pregnancy or early at postpartum reported results by BMI. In a study by Wei et al. in South Carolina, US, women had DXA scans at 12–20 weeks of gestation and 0–14 weeks postpartum; women with obesity had higher femoral neck and spine BMD at both gestation and postpartum visits. However, obesity was associated with greater loss of femoral neck BMD during pregnancy (p < 0.001).^37^ In Japanese women, Yoshikata et al. preformed calcaneus quantitative ultrasound during pregnancy and DXA postpartum; researchers found that first trimester BMI was positively correlated with BMD in all weight categories after delivery stages (r = 0.49 at delivery, r = 0.54 at 6 months postpartum, and r = 0.47 at 1 year postpartum).^38^ Although our results cannot be directly compared to those studies since we did not measure BMD, our findings are in line with our hypothesis of higher BMD reflected in lower Z-SOS scores. They are also in line with suggesting important changes in BR over the stages of pregnancy in relation to pregnancy weight. Also, we collected distal radius measurements, a predominantly trabecular type of bone undergoing more metabolic demands during pregnancy, thus limiting our ability to compare to studies that measured predominantly cortical bone.

Although specific for BMD evaluation, the use of DXA during pregnancy is limited due to exposure to ionizing radiation to both mother and fetus. Therefore, other techniques measuring components of bone structure such as axial ultrasonographic studies of appendicular bones and urinary biomarkers have been used.^39^ Bone mass reaches its peak during adulthood and, in women, pregnancy is one of the most important factors that can affect bone metabolism and remodelling.^40^ The observed reduction in the speed of sound z-scores, among individuals with higher BMI categories, stands in contrast to the lack of significant differences in markers of bone formation (alkaline phosphatase) and resorption (N-telopeptides). This apparent contradiction underscores the complex relationship between adiposity, bone turnover, and bone quality.

Speed of sound (SOS) reflects bone quality and structure, not just mineral content.^25,41-43^ It's possible that overweight and obese individuals experience changes in bone microarchitecture (e.g., reduced trabecular connectivity or cortical thickness) rather than turnover rates. These structural changes can result in weaker bones without corresponding changes in formation or resorption markers. Additionally, obesity may influence the mineral-to-collagen ratio or bone matrix quality.^44^ For instance, the accumulation of advanced glycation end products (AGEs) in collagen could compromise bone strength without altering resorption or formation processes.^45^ While bone turnover markers reflect systemic formation and resorption activity, they do not capture changes in bone microarchitecture or material composition. Obesity may thus impair bone strength by adversely affecting the bone's microstructure, including trabecular connectivity and cortical thickness. Such structural alterations, which can reduce bone quality, may occur without detectable changes in systemic turnover markers. Furthermore, obesity-related changes in bone material properties, such as the accumulation of advanced glycation end-products (AGEs) in collagen, could weaken bone without altering turnover dynamics.

From a mechanical perspective, increased body weight in obese individuals imposes greater mechanical loading on the skeleton, which theoretically should stimulate bone formation. However, this benefit may be offset by maladaptive remodelling due to chronic excessive loading, particularly in trabecular bone.^46^ For example, localized stress in obese individuals might exceed the bone's capacity for adaptive remodelling, leading to structural compromise. Additionally, fat infiltration into the bone marrow—a phenomenon observed in obesity—may disrupt osteoblast function and lead to impaired bone formation.^47^

Endocrine and metabolic factors provide further insight into this relationship. Obesity is characterized by an altered adipokine profile, including elevated levels of leptin, reduced adiponectin, and increased pro-inflammatory cytokines (e.g., TNF-α and IL-6).^48^ Chronic inflammation and hormonal dysregulation can impair osteoblast activity and promote osteoclastogenesis, potentially tipping the balance toward bone loss or compromised quality.^49^ Concurrently, the sequestration of vitamin D in adipose tissue reduces its bioavailability, impairing calcium absorption and mineralization.^50^ This subclinical vitamin D deficiency, common in obese individuals, may contribute to decreased bone strength without immediate effects on turnover markers.

Gestational-specific factors may also play a role. Changes during pregnancy, such as elevated progesterone and relaxin, may further influence bone remodeling, potentially affecting obese individuals differently than their normal-weight counterparts.^51^

A strength of our study is the longitudinal follow-up, measuring Z- SOS score and urinary NTx in every pregnancy trimester, enabling us to analyse trends and changes of bone turnover during an entire BR cycle adjusting for both fixed and time-varying covariables. We observed that pregnant women with normal weight had a slight increase in Z- SOS scores from first to second trimester and a significant decrease from second to third trimester. These results show an expected pattern of complete BR during pregnancy, characterized first by an increased formation of organic matrix and later by a more pronounced mineralization process of the organic matrix that physiologically compensates for the higher demands of mineral mobilization to meet fetal requirements. This pattern of increased mineralization at third trimester is confirmed by our NTx values that increased as pregnancy advanced in all BMI groups. Previous studies, using DXA, ultrasonographic methods, and bone turnover biomarkers, have shown similar results in BR during pregnancy. Aguado et al. showed that velocity of sound propagation was lower during the second and third trimester of pregnancy in comparison to first trimester (p< 0.0001),^52^ and Kraemer et al. measured ultrasonometry of the heel and observed a reduction in the median of Z-SOS scores from the first measurement (weeks 10–22 of gestation) to follow up after delivery (0–9 days).^53^ In a cohort study, Moller et al. reported that urinary NTx increased from early pregnancy and remained higher throughout pregnancy, particularly from second to third trimester (p < 0.001).^54^

Our models accounted for important potential confounders such as weight gain at each pregnancy trimester, multiparity, total breastfeeding, and intake of specific nutrients. Approximately 80% of the calcium, phosphorus, and magnesium required for foetal development are obtained during third trimester of pregnancy and several physiological mechanisms are used to meet these needs. Increased intestinal absorption compensates for the increased requirements.^34^ However, if dietary recommendations are not fulfilled and calcium and phosphorus intake are insufficient, a greater increase in bone resorption will occur. This may be particularly important in pregnant women with unhealthy dietary patterns characterized by an increase in consumption of energy dense, low-nutrient foods.

A limitation of this study is that we could not control for physical activity and, therefore, we cannot adjust for any effect of exercise on BR. However, it is not expected that pregnant women, particularly late in pregnancy, would perform moderate to high impact physical activity which is typically the kind of exercise that contributes to a higher bone remodelling.^55^ Another limitation is that our study included relatively healthy pregnant women, which may have attenuated the effects of other metabolic conditions in pregnancy such as hypertension, preeclampsia, or gestational diabetes. From a clinical point of view, since pregnancy is a state of high mineral demand that is met from the maternal skeleton (which increases remodeling), if there is in fact an impact on BMD it would be advisable to follow-up to study and correct the deficiency. However, full-body DXA scans including the spine and hip at the 4th month and one year postpartum would be ideal however these are not commonly performed.

## Conclusion

Bone remodelling patterns across pregnancy trimesters, as assessed by SOS measurements and bone turnover markers, differ in women who are overweight or obese as compared to normal weight women. This study highlights the need to distinguish between bone turnover and bone quality in understanding the skeletal effects of obesity. The observed reduction in bone speed of sound among obese individuals likely reflects a multifactorial process involving altered microarchitecture, endocrine dysregulation, and localized remodeling adaptations. Future research should focus on advanced imaging techniques, such as high-resolution peripheral quantitative computed tomography (HR-pQCT), to assess bone microarchitecture. Additionally, integrating measurements of adipokines, inflammatory markers, and vitamin D status could provide a more comprehensive understanding of the interplay between obesity and bone health. Addressing these knowledge gaps will be critical for developing targeted interventions to mitigate the adverse skeletal effects of obesity, particularly during pregnancy. Lastly, follow-up of these women will provide insight to the long-term relation between pregnancy and midlife bone health.

## Figures and Tables

**Table 1.** Descriptive statistics at baseline in women that had at least one N-telopeptides measurement during pregnancy (N=579).

	Normal BMI			Overweight			Obese			Overall			
Variable	n	Mean	SD	n	Mean	SD	n	Mean	SD	n	Mean	SD	p-value

Gravidity													
Primigravida	114	43%		72	32%		20	23%		206	36%		0.002
Multigravida	154	57%		153	68%		66	77%		373	64%		

N-Telo nM BCE per mM Creatinine	266	77.13	65.52	225	70.09	45.42	85	65.67	36.07	576	72.69	54.69	0.150
Bone Specific Alkaline Phosphatase (ng/ml)	41	12.5	4.02	44	12.69	4.36	15	12.93	3.27	100	12.65	4.04	0.816
Radius speed of sound Z-score	126	−0.12	1.03	128	−0.18	1.1	39	−0.29	1.12	293	−0.17	1.07	0.418
Age (years)	268	25.37	5.44	225	26.79	5.25	86	28.74	5.33	579	26.42	5.47	0.000
Body Mass Index kg/m^2^	268	22.56	1.77	225	27.17	1.28	86	32.52	2.31	579	25.83	3.9	0.000
Number of pregnancies	268	1.97	1.05	225	2.05	0.94	86	2.4	1.21	579	2.06	1.04	0.008
Total lifetime months of breastfeeding	267	6.47	9.86	225	6.13	8.56	86	7.17	8.71	578	6.44	9.2	0.186
Total years of schooling	267	10.67	2.73	225	10.92	2.99	86	10.03	3.09	578	10.67	2.9	0.255
Age at 1st pregnancy (years)	268	21.75	4.8	225	22.18	4.84	86	21.99	4.29	579	21.96	4.74	0.450
Total Daily Calcium Intake (mg)*	268	1125.16	539.86	225	1059.63	514.56	86	1076.06	455.45	579	1092.4	518.37	0.328
Daily Dietary Vitamin D (mg/day)	268	33.26	34.86	225	32.11	31.79	86	29.69	30.77	579	32.28	33.07	0.516
Daily Dietary Phosphorous (mg/day)	268	1425.29	546.93	225	1330.09	516.69	86	1351.36	427.06	579	1377.31	520.1	0.100
Daily Dietary Manganese (mg/day)	268	320.45	115.1	225	301.54	116.49	86	321.5	106.69	579	313.26	114.63	0.070
Weight (kg)	268	53.91	5.55	225	64.41	5.83	86	77.93	7.34	579	61.56	10.27	0.000
Weight gain (kg)	242	9.72	3.31	208	8.17	4.02	75	7.09	3.54	525	8.73	3.76	0.000
Gestational age (weeks)	268	13.3	1.71	225	13.23	1.79	86	13.62	1.36	579	13.32	1.7	0.076

* Including calcium from supplement

**Table 2.** Mixed effects models with random intercepts: Longitudinal association of BMI categories at first trimester and radius Z-scores, urinary N-telopeptides and bone specific alkaline phosphatase in blood, during pregnancy. Normal BMI was the reference category in all models. Models adjusted for age at recruitment, education, age at first pregnancy, number of pregnancies, total breastfeeding, pregnancy weight gain at each trimester visit, total calcium intake, dietary intake of phosphorus, magnesium, and vitamin D.

Outcome	Observations	Women	BMI Category	b	D%	95% CI	p-value

N-Telo nM BCE per mM Creatinine	1629	577					
			Overweight		−2.49	(−9.55, 5.11)	0.510
			Obese		−3.73	(−13.43, 7.06)	0.483
Bone Specific Alkaline Phosphatase (ng/ml)	354	143					
			Overweight		0.76	(−9.92, 12.70)	0.895
			Obese		−3.67	(−17.82, 12.91)	0.645
Radius Speed of Sound Z-Score	922	414					
			Overweight	−0.28		(−0.49, −0.08)	0.007
			Obese	−0.57		(−0.87, −0.27)	0.000
